# Supplementary material for: Emission Enhancement of Ge/Si Quantum Dots in Hybrid Structures with Subwavelength Lattice of Al Nanodisks
Source: Nanomaterials (Basel). 2023 Aug 25;13(17):2422. doi: 10.3390/nano13172422 (PMC10490227; doi:10.3390/nano13172422)
Supplement: Supplementary file 1 [file nanomaterials-13-02422-s001.zip › nanomaterials-2559718-supplementary.pdf]

nanomaterials

MDPI

Supporting Information

Article

Emission Enhancement of Ge/Si Quantum Dots in Hybrid Structures with Subwavelength Lattice of Al Nanodisks

Vladimir A. Zinovyev [1,*], Zhanna V. Smagina [1], Aigul F. Zinovieva [1,2], Aleksei A. Bloshkin [1,2], Anatoly V. Dvurechenskii [1,2], Ekaterina E. Rodyakina [1,2], Margarita V. Stepikhova [3], Artem V. Peretokin [3] and Alexey V. Novikov [3]

[1] Rzhanov Institute of Semiconductor Physics, Siberian Branch of Russian Academy of Sciences, 630090 Novosibirsk, Russia; smagina@isp.nsc.ru (Z.V.S.); aigul@isp.nsc.ru (A.F.Z.); bloshkin@isp.nsc.ru (A.A.B.); dvurech@isp.nsc.ru (A.V.D.); rodyakina@isp.nsc.ru (E.E.R.)
[2] Department of Physics, Novosibirsk State University, 630090 Novosibirsk, Russia
[3] Institute for Physics of Microstructures of the Russian Academy of Sciences, 603950 Nizhny Novgorod, Russia; mst@ipmras.ru (M.V.S.); aperetokin@ipmras.ru (A.V.P.); anov@ipmras.ru (A.V.N.)
* Correspondence: zinoviev@isp.nsc.ru

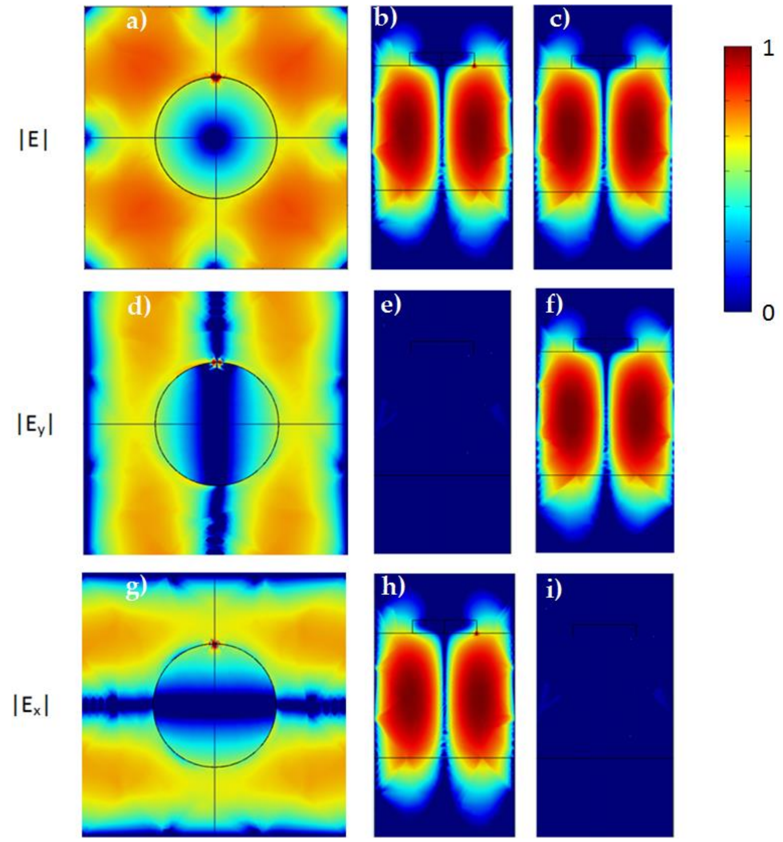

Figure S1. Near field distributions calculated at λ=1.742 μm for the model structure that is an analog of the experimental SOI structure with a square lattice of Al disks on the top of it (D = 250 nm and a = 540 nm). XY-sections (a,d,g) are taken at the interface air/Si. XZ-sections (b, e, h) and YZ-sections (c, f, i) cross the center of Al disk. Point dipole oriented along x-direction is placed under the edge of Al disk at the depth 10 nm. Here z-component of electric field is not shown, because $|E_z|$ = 0. The color scale is shown in the right.

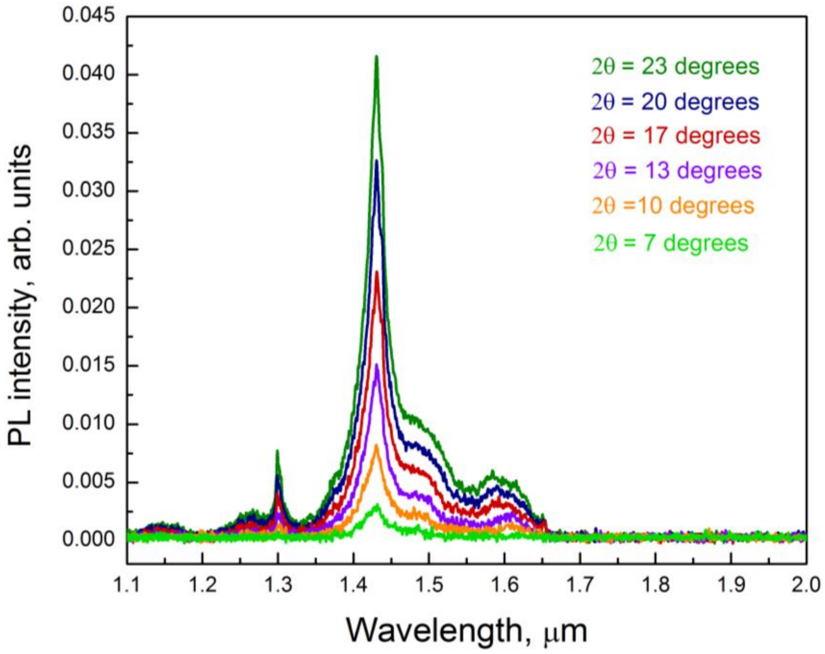

Figure S2. The dependence of micro-PL spectra of the structure on the collection angle 2θ at fixed disk diameter D = 250 nm and lattice period a= 540 nm. Excitation laser wavelength 532 nm, power 20 mW. The temperature of measurement 300 K.

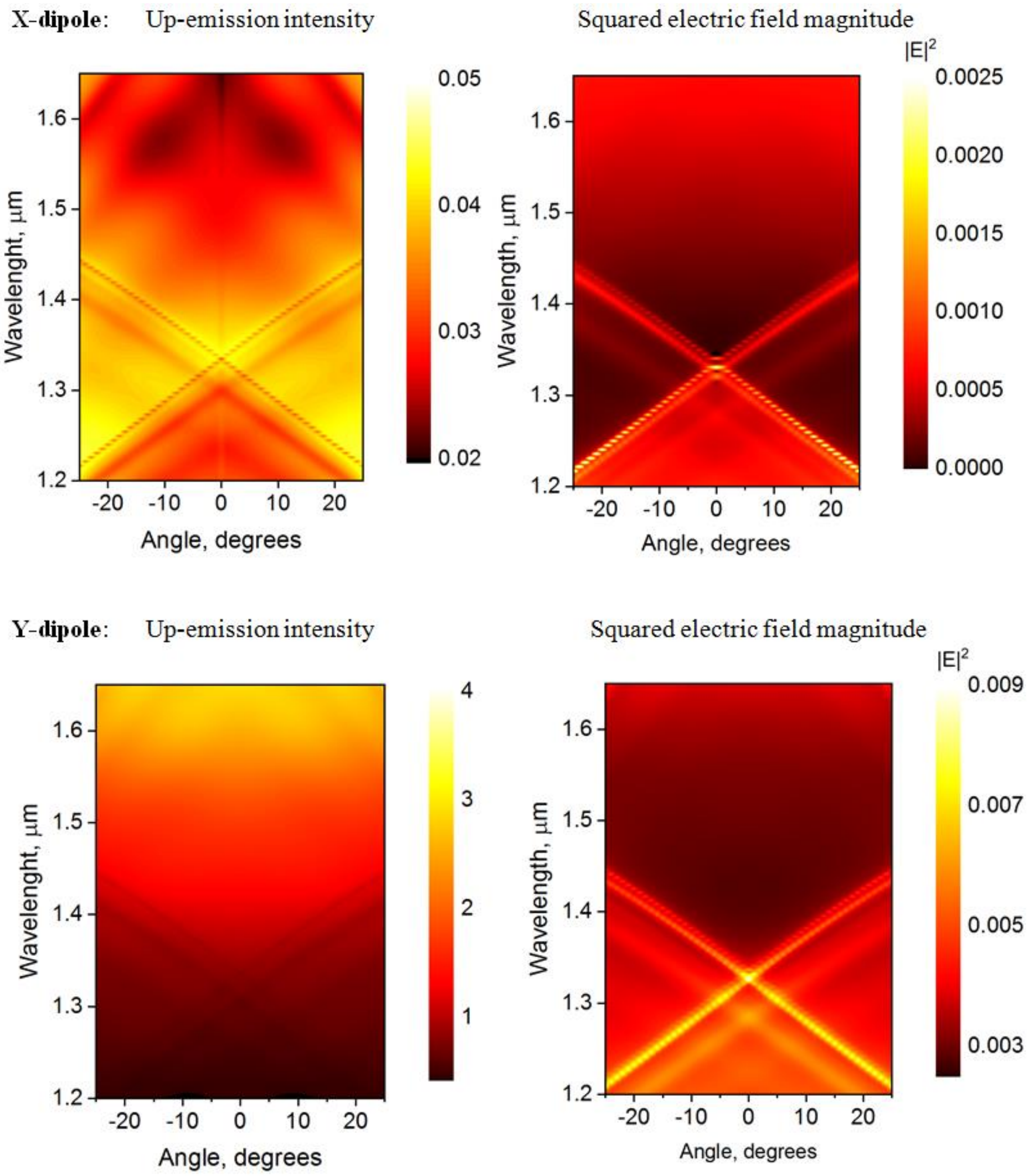

Figure S3. Up-emission intensity (left) and squared electric field magnitude (right) dependences on the radiation exit angle calculated in wavelength range from 1.2 to 1.65 μm for plasmonic structures with square lattice of Al disks on semi-infinite Si substrate (disk diameter D = 250 nm and period a = 540 nm). Top panels demonstrate the dispersion dependence of the X-dipole emission. Bottom panels show the dispersion dependence of the Y-dipole emission.

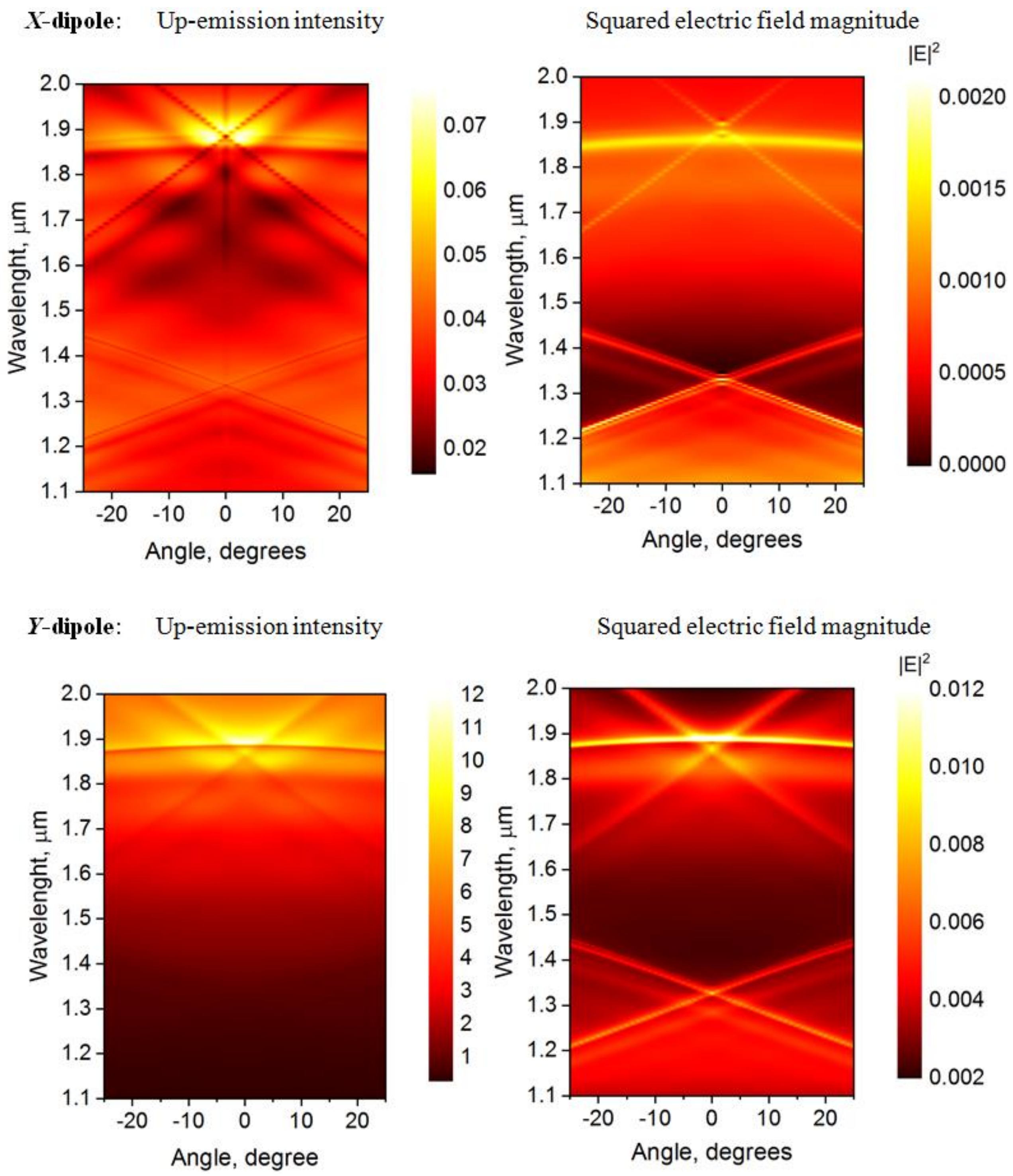

Figure S4. Up-emission intensity (left) and squared electric field magnitude (right) dependences on the radiation exit angle calculated in extended wavelength range from 1.1 to 2 μm for plasmonic structures with square lattice of Al disks on semi-infinite Si substrate (disk diameter D = 250 nm and period a = 540 nm). Top panels demonstrate the dispersion dependence of the X-dipole emission. Bottom panels show the dispersion dependence of the Y-dipole emission.

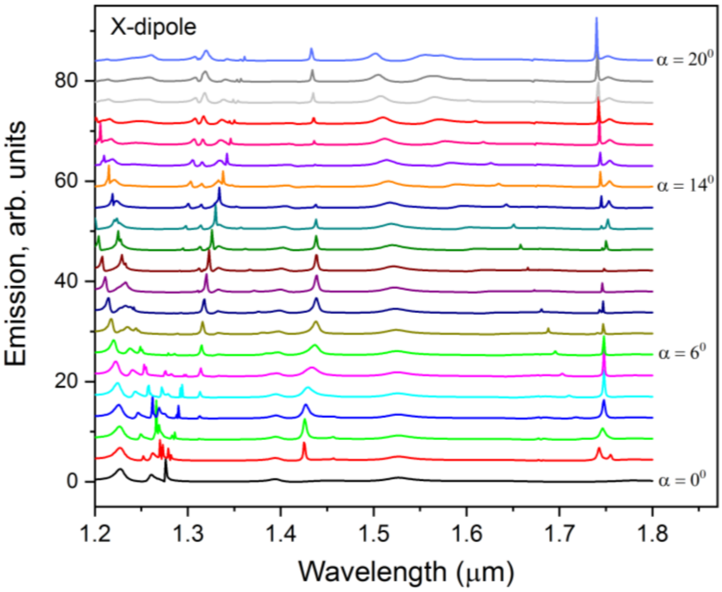

Figure S5. The emission spectra of the model structure with a square lattice of Al nanodisks created on the top SOI structure at different radiation exit angle α. The emission spectra are calculated for the point dipole oriented along x-direction placed under the Al disk edge at the depth 10 nm. The diameter of the disks *D* = 250 nm, the lattice period *a* = 540 nm.

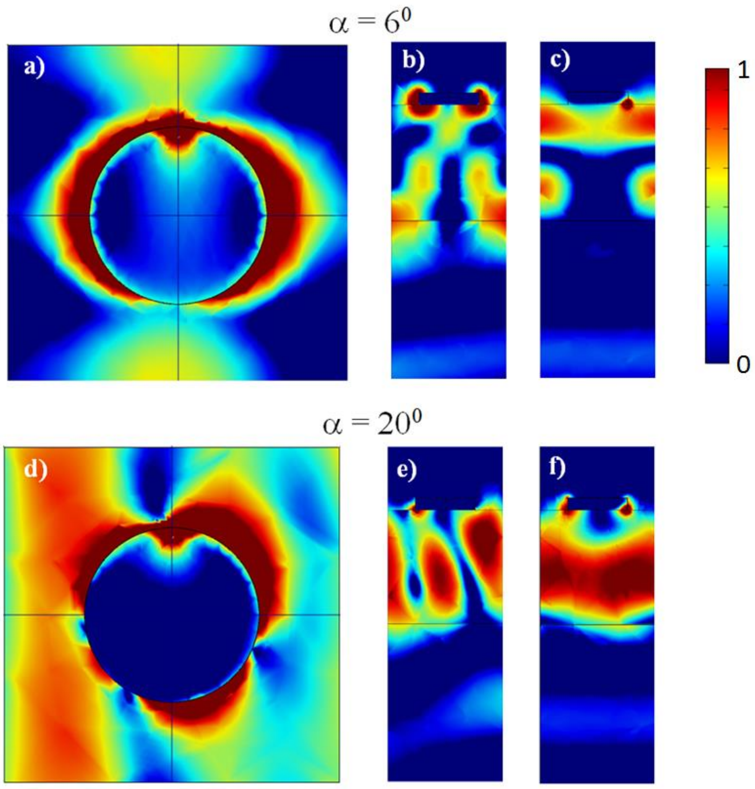

Figure S6. Near field distributions calculated for the hybrid structure with a square lattice of Al disks coupled to a Si waveguide layer (disk diameter *D* = 250 nm and period *a* = 540 nm) at radiation exit angle α = 6° at λ =1.437 μm (top panels) and α = 20° at λ =1.433 μm (bottom panels). *XY*-sections (a, d) are taken at the interface air/Si. *XZ*-sections (b, e) and *YZ*-sections (c, f) cross the center of Al disk. Point dipole oriented along x-direction is placed under the edge of Al disk at the depth 10 nm.
